# Supplementary material for: A critical-like collective state leads to long-range cell communication in Dictyostelium discoideum aggregation
Source: PLoS Biol. 2017 Apr 19;15(4):e1002602. doi: 10.1371/journal.pbio.1002602 (PMC5396852; doi:10.1371/journal.pbio.1002602)
Supplement: S1 Appendix — Includes detailed explanation of experimental procedure, single-cell and coarse-grained models, and experimental data analysis. (PDF) [file pbio.1002602.s001.pdf]

Supporting Information

Giovanna De Palo[1,2], Darvin Yi[3,4,5], and Robert G. Endres[1,2]

[1]Department of Life Sciences, Imperial College, London, United Kingdom
[2]Centre for Integrative Systems Biology and Bioinformatics, Imperial College, London, United Kingdom
[3]Joseph Henry Laboratories of Physics, Princeton University, USA
[4]Lewis Siegler Institute for Integrative Genomics, Princeton University, USA
[5]current address: Department of Radiology, School of Medicine, Stanford University, USA

Contents

1 Experimental procedure **2**

2 Primer on spatial information **2**

3 Single-cell model **3**

4 Coarse-grained model **4**

5 Experimental data analysis **7**

6 Mutant simulations and comparison with data **8**

1 Experimental procedure

To capture the data, fluorescence microscopy was performed using a Nikon TE300 microscope with a Xenon lamp light source. Cultures of axenic *Dictyostelium discoideum* expressing mRFPmars (TRED marker) [1] and the Epac1camps FRET sensor [2] were starved for 4-5 hours, counted in a hemocytometer and mixed in a 1:9 ratio. Appropriate volumes to achieve the 10% TRED fraction were plated on glass. To ensure a flattened 2-dimensional image, the cells were then covered with a sheet of 1% ultra-pure agarose measuring 0.5mm in thickness. To cover a larger field of view, 16 images were taken in a 4x4 grid, each image capturing an area of 300μm x 300μm for a total field of view of 1.2mm x 1.2mm. At each position, three fluorescent images were taken: an image to detect the ECFP component of the Epac1camps molecule, an image to detect the EYFP component of the Epac1camps molecule, and a Mars Red component to detect the cells expressing mRFPmars. Exposures for the ECFP and EYFP images were 20ms while the exposure for the Mars Red image was 100ms. Images were taken with a period of around 45 seconds. The FRET signal, which is proportional to cytosolically produced cAMP, is calculated by the negative ratio of the EYFP and ECFP fluorescence intensities of the respective acceptor and donor fluorophores, corrected for photobleaching and cytosolic pH changes. For further details see [2, 3].

2 Primer on spatial information

Shannon's concept of entropy was originally applied to telecommunication and broadcasting to understand the principles and constraints of information transmission [4, 5]. Suppose we have a random variable x, produced by a random but nevertheless well-defined process. We ask how much uncertainty do we have prior to receiving the outcome, such as a particular value for x. The amount of uncertainty can also be regarded as the 'degree of surprise' on learning the outcome. For the outcome of an improbable event we are very surprised, and we were uncertain to begin with. In contrast, for a very probably result we are little surprised and we were also little uncertain at the start.

A general measure of uncertainty (or entropy) $h(x)$ should therefore depend on the probability of observing x. Specifically, it should depend monotonically on the probability $p(x)$. Furthermore, the information of observing two independent events x and y should add up $h(x, y) = h(x) + h(y)$. Since for such events $p(x, y) = p(x)p(y)$, the logarithm has the desired monotonicity and additivity properties. Specifically, we use

$$h(x) = -\log_2 p(x), \tag{S1}$$

where the negative sign ensures that the entropy is positive. The choice of the base 2 of the logarithm is arbitrary but provides the entropy in units of bits. The uncertainty of one bit corresponds to a choice of two possible, equally likely outcomes for a random variable.

To calculate the average amount of entropy over several trials of transmitting outcomes, we average $h(x)$ over the probability distribution $p(x)$ and obtain Shannon's entropy formula

$$H[x] = -\sum_x p(x) \log_2 p(x). \tag{S2}$$

To show that the definition makes sense, let us consider a couple of simple examples. First, let us assume that one outcome x_0 occurs with absolute certainty, so that $p(x_0) = 1$ and $p(x \neq x_0) = 0$. Hence, both the former, $1 \log(1) = 0$, and latter, $0 \log(0) = 0$, contribute no entropy (uncertainty). Hence, as the outcome is clear, there is no entropy to start with. Second, consider a Gaussian distributed random variable, $p(x) = 1/\sqrt{2\pi\sigma^2} \exp\{-(x-\mu)^2/(2\sigma^2)\}$ with average μ and variance σ^2. This distribution has the entropy value $H = 1/2\{1+\ln(2\pi\sigma^2)\}$. Hence, as expected, the narrower the distribution, the smaller the entropy.

We can now use the concept of Shannon's entropy to define 'information', or spatial information of a cell distribution in our case. The spatial information SI can be defined as the entropy of a maximally disordered (maximum entropy) cell distribution minus the entropy of the actually, potentially peaked cell distribution

$$SI = H_{\text{max}} - H_{\text{actual}}. \tag{S3}$$

Hence, the more ordered (peaked or structured) the actual cell distribution, the higher the spatial information as the entropy (uncertainty about where cells are) decreases with increasing order (S1 Fig). To apply this concept to two-dimensional (2D) images, it is convenient to define this spatial information in k-space (or Fourier space) using a superposition of waves with different amplitude and wave length, leading to the formulas provided in the Materials and Methods section of the main text (see also [6] for a formal derivation).

This spatial information can also be thought of as a measure of the confidence to predict the presence (or absence) of a cell at a given point on the 2D surface (see S2 Fig for details). Alternatively, compression algorithms can be applied to images to mimic spatial information as illustrated by applying GZip to JPG data images (S3 Fig). Image compression reduces uncertainty by identifying and eliminating statistical redundancy in an image, resulting in a smaller file size.

3 Single-cell model

To reproduce the dynamics of single *Dictyostelium* cells, we extended a detailed model previously designed in our group [7]. In this model the cell membrane is represented by a contour evolving according to the Meinhardt model [8]. Three interacting molecular species are modeled by means of partial differential equations numerically solved along the 1D cell contour: a local activator a, a global inhibitor b and a local inhibitor c. The dynamics of these equations depend on sensing of extracellular cAMP concentration by means of a "chemosensation" contribution, calculated by a sum of three terms: the actual environmental stimulus, Poisson-like noise, and an auto-activation term. The movement of the contour of the cell was determined by the force acting on the membrane. This was calculated by subtracting cytosolic pressure and membrane tension from the outward force proportional to the concentration of the local activator a (for details see Supplementary Information of [7]).

Intra- and extracellular dynamics of cAMP were added to this model, along with the intracellular (extracellular) dynamics of RegA (PDE), responsible for degrading cAMP. In particular, to reproduce the cAMP dynamics we adopted the FitzHugh-Nagumo model [9, 10]. Two ordinary differential equations represent the concentrations of intracellular cAMP and intracellular phosphodiesterase RegA:

$$\begin{aligned} \frac{dcAMP}{dt} =& k_1[(cAMP - cAMP_0) - \frac{1}{3}(cAMP - cAMP_0)^3 \\ &- RegA + k_2] + k_3 \cdot S - cAMP_l \end{aligned} \tag{S4}$$

$$\frac{dRegA}{dt} = \frac{1}{k_1}(k_4 \cdot cAMP - k_5 \cdot RegA - k_6 \cdot S). \tag{S5}$$

The input sensation S is calculated as the maximum amount sensed by a node along the contour of the cell. In Eq. S4, $cAMP_0$ is set as a reference threshold, such that the cell is pulsing if the term $(cAMP - cAMP_0) > 0$, therefore secreting in the environment at a rate proportional to $\frac{1}{3}(cAMP - cAMP_0)^3$. If instead the concentration of intracellular cAMP is smaller than $cAMP_0$, the cell is just leaking cAMP at a rate proportional to $cAMP_l$, assumed to be constant.

The secretion of cAMP occurs at the rear of the cell [11, 12]. In order to simulate this secretion, cAMP is added to the environment grid in proximity to the contour points opposite to the direction of motion with the greatest membrane curvature. PDE is also released into the environment at a constant

rate to simulate the secretion of the specific phosphodiesterase DdPDE1 [13], resulting in

$$\frac{dcAMP_{ex}}{dt} = -k_7 \cdot cAMP_{ex} - k_8 \cdot PDE + D_1 \nabla^2 cAMP \tag{S6}$$

$$\frac{dPDE}{dt} = -k_9 \cdot PDE + D_2 \nabla^2 PDE \tag{S7}$$

In addition to diffusive and degradation terms for extracellular cAMP and PDE, the concentrations of secreted cAMP and PDE are also added to the environment at the corresponding grid points. Both extracellular cAMP and PDE are free to diffuse in the environment without absorption by cells. Parameter values are provided in S1 Table. After pulsing, a cell undergoes a refractory period, lasting about 400 s during which the cell cannot repolarize. Furthermore, during the following 400 s the cell can repolarize but cannot secrete pulses of cAMP.

4 Coarse-grained model

Model description. The coarse-grained model is designed to reproduce the behavior of an entire cell population based on a minimal set of single-cell features. Cells are represented as point-like objects, and free to move in space, without any spatial grid. This avoids discretizing their position and the concentration of cAMP in the environment.

This coarse-grained model keeps the main features and results of our detailed model, while being much faster and computationally less expensive. In particular, the concentration of cAMP secreted by a single cell, when pulsing or leaking, was extracted from the detailed simulations. In this way the partial differential equations representing diffusion of cAMP and its degradation by PDE can be excluded from the computation. The spatial cAMP profile was extracted by considering the spatial concentration of leaked or pulsed cAMP at the rear of the cell in the direction opposite to the cell motion. More specifically, an exponential profile was used, with maximal value and time dependence derived from detailed simulations. A spatial decay constant of 0.1 μm^{-1} was chosen, slightly different from the constant of 0.2 μm^{-1} estimated from the detailed model. This choice allowed us to introduce volume exclusion for wider streams of cells when aggregating, closer to the experimental results. See S4 Fig for a comparison of the secretion profiles.

To simulate secretion at the rear of the cell, secretion is maximal in the direction opposite to the direction of motion. For the other directions we rescaled the amount of secretion according to the cosine of the angle between the direction opposite to motion and direction considered. If this angle is greater or equal to $\pi/2$, secretion is set to zero. The physical cell dimension is considered as follows: the secretion profile is set to the maximum value within a radius of 10-13 μm from the cell centre, rescaled according to the direction of motion. Knowing the positions and spatio-temporal secretion profiles of each cell, we were able to estimate the amount of cAMP that every cell senses and the gradient of the cAMP concentration they experience. To these concentration values, we added Poisson-like sensing noise as done for the detailed model. Furthermore, cAMP leakage increases linearly with development time for every cell, up to a maximum reached when the cells become "movement competent" (discussed next; all cells stop increasing cAMP leakage at most after 10,000 seconds $\simeq$ 2.8 hours).

To represent the movement by pseudopod extension (with angle $\simeq$ 55°between old and new pseudopods [14]), we allow the cells to move in just two possible directions, each forming an angle of ±27.5°relative to the previous direction of motion. When the cAMP concentration is low, cells move by randomly choosing between these two possible directions. When instead the cell becomes "movement competent" it chooses the closest-to-best direction according to cAMP gradient from the two available.

As shown in the schematic of S5 Fig, when cells sense a cAMP concentration above a certain threshold c_1 (equal to 1200), they "spike", emitting the cAMP concentration as estimated for a pulse from the

single-cell model over the same time course. After pulsing cells enter a refractory period that lasts for 6 minutes in line with experiments [2]. A second threshold ∇c_2 (equal to 200) characterizes cell movement: when a cell perceives a cAMP gradient greater or equal ∇c_2, it switches to the "movement competent" state, in which it moves according to the concentration of cAMP in the environment (and leakage is not increased any further). Once a cell is "movement competent", it remains polarized during the refractory period, thus keeping the same direction of motion for half of the refractory period (again mimicking the single-cell behavior of the detailed model). Finally, cell speed is set equal to 25 nm/s, in line with the speed we estimated from our experimental data.

We also introduced a minimal cell-to-cell distance of 3 μm to avoid complete overlapping of pairs of cells. This volume exclusion condition was relaxed at later stages of aggregation, considering high density when space was completely filled inside a neighborhood radius of 18 μm around the cell.

Cells are initially uniformly distributed in space and move randomly, without secreting cAMP, for the first 100 iterations (about 7 minutes). Cell density is taken from experimental data and set to $6.6 \cdot 10^{-3}$ cells/μm^2 for simulations of N=1000 cells. Space dimension (L=389 μm) was then fixed for simulations with N=1200, 800 and 600 cells.

Estimating connected correlations. The method for estimating the connected correlations, described in the main text in the Directional correlations and susceptibility section of *Materials and methods*, is reported in more detail in S6 Fig. Briefly, instead of subtracting the global average to calculate the fluctuations in direction (panel A) [15], we subtract the local averages (B), thus avoiding an overestimation of the correlations.

Perturbations and 'steering'. Here, we considered the dynamics of aggregation more closely. When increasing the density of cells in our simulations, we noticed that cells aggregate faster at higher cell densities as measured by the slope of the spatial information (S7 Fig A). This increase in speed appears to reflect the increased ability to relay the signal as nearby cells can become excited and pulse themselves, facilitating aggregation. To test this prediction, we attempted to quantify the speed of aggregation in our time-lapse movies as well. Unfortunately, the experimental movies were too noisy or variable to confirm this trend (S7 Fig B; the dark blue dashed curve violates the trend).

Critical-like behavior is the tipping point between order and robustness on one side and disorder and chaos on the other side. This point may highten the sensitivity of the collective to detect changes in external cAMP concentration and to help cells make decisions on when and where to aggregate. Here, we attempted to investigate this with our simulations. For this purpose, we applied both local and global cAMP perturbations to our system of cells; local perturbations represent a short local pulse of cAMP released on the 2D surface, while a global perturbation represents an overall pulse of cAMP on the whole area of observation. The localized cAMP instead was simulated considering 2D diffusion of a point-like source equation:

$$cAMP(r,t) = c_0 \frac{e^{-\frac{r^2}{4Dt}}}{4\pi Dt}$$

with r being the distance from the source and D representing the diffusion coefficient of cAMP (250 $\mu m^2/s$) [16, 17]. The source position was chosen randomly with $c_0 = 5 \cdot 10^4 (4\pi D)$ to keep the initial amplitude equal to global perturbation.

These perturbations were applied at two different time points, during prestreaming, i.e. after $2,000$ s ($\sim$33 min) of the start of simulations, and during streaming, after $6,200$ s ($\sim$103 min). The global release had a peak amplitude of $5 \cdot 10^4$ (for units see caption of Table **??**) and an exponential decay in time with a constant of 0.025 s^{-1}. Results are shown in movies S6-S9. In these movies, the pulse timing (and position in case of local perturbations) are shown by a yellow asterisk. For local perturbations, a colormap is also used underneath the cells to represent the external concentration of cAMP. Note that

these simulations deal with cAMP propagation based on diffusion of cAMP (with diffusion coefficient of 250 $\mu m^2/s$). Consequently, cAMP propagates much faster than cell movement with maximal speed of 0.5 $\mu m/s$ [18].

Whether the perturbations were applied before and during streaming had a noticeable different effect on aggregation. A local perturbation prior to streaming induces aggregation at the location of the pulse, although the aggregation centre may shift later during the simulation. A global perturbation prior to streaming has a similar affect, although the location of aggregation cannot be influenced. In contrast, applying such perturbations during streaming has largely no effect. While local perturbation may somewhat influence the location of aggregation, the overall dynamics stay the same. Indeed, comparing the time courses of the spatial information, we notice that the shape of the curve always stays the same, and only the onset of aggregation can be shifted to earlier times with local or global cAMP perturbations (S7 Fig C). In particular, early local perturbations allow the possibility of steering cells to locations of aggregation.

Comparison with recent analyses and models. Model and experimental data presented in this manuscript were compared with recently published models and analyses, which also focused on *Dictyostelium* cell populations and the FitzHugh-Nagumo model for the excitatory cell behavior [2, 3, 19]. S8 Fig shows how oscillations in intracellular concentration of cAMP are captured by our coarse-grained model (panels A and B), and we successfully reproduce spatial propagation of cAMP waves (panels C and D) [2]. Moreover, similar to the FitzHugh-Nagumo model described in [3], in our detailed model the single-cell adaptation time in response to step changes of cAMP increases with increasing input concentration (S9 Fig A). The external cAMP rate also plays an important role in shaping the response of single cells. In fact, as shown in [3], responses to step changes are different to responses to exponential ramps of extracellular cAMP, even though the final maximal concentrations are the same. This behavior changes in the case of high concentrations of cAMP. S9 Fig B shows the responses of our detailed model, while S9 Fig C shows the corresponding results of our coarse-grained model.

5 Experimental data analysis

In this section we present additional details and results from the image analysis of the experimental movies considered in the main text.

Estimating fraction of TRED cells. As previously explained, a subpopulation of cells was imaged expressing the TRED marker. This allowed us to distinguish single cells even in high-density movies and to track their movement. In order to understand the effect of cell number on critical behavior, it is important to estimate the total number of cells in the experiments, and to make sure that the fraction of TRED cells is the same for the different movies, thus avoiding artifacts in the evaluation of connected correlations. The TRED fraction was estimated by considering the area covered by TRED cells and comparing this area with the area occupied by the whole cell population, estimated from the CFP images. For the first 200 frames, individual TRED and CFP images were pretreated with MATLAB function *imadjust*, which expands the range of intensities of the image such that the dimmest (brightest) 1% of the total pixels are saturated at low (high) intensities. After this, *graythresh* and *im2bw* functions were applied in order to create a mask separating cell and non-cell pixels, choosing the intensity threshold, which minimizes the intraclass variance of black and white sets. Results for the number of tracked TRED cells and the TRED/CFP cell-area ratio are presented in S10 Fig. We considered the peak of the TRED number during the streaming phase for determining the number of cells reported in the legend of Fig 3D, of the main text. For dataset 6 (represented in yellow), since the ratio of TRED cells is consistently higher (see S10 FigB), we randomly down sampled the number of TRED cells, considering about half of the tracked cells. Hence, we obtained a TRED fraction similar to the other datasets.

Further evidence of criticality. Here, we illustrate further features of the experimental system, consistent with criticality, using dataset 3 as an example. We first considered the speed of cells, estimated from the average movement per frame of all the tracked cells (see S11 Fig A). Cell speed is not constant during aggregation; instead a slowing down of cells is clearly noticeable before the streaming phase, which is a common signature of critical systems [20]. Note the oscillations in speed during the first part of the movie. If we compare these oscillations with the ones present in the FRET signal we can notice that they have the same period but are anticorrelated (see S11 Fig B). Since the FRET signal is low when intracellular cAMP in high and vice versa, these results suggest that cells slow down when secreting cAMP, and that they speed up when accumulating cAMP internally.

When dealing with criticality, the increase of correlations in fluctuations is often accompanied by a departure from a Gaussian distribution of these fluctuations [21]. We thus considered the distribution of the different angles of these fluctuations and compared them with the von Mises distribution (i.e. the Gaussian probability distribution on a circle), generated by using MATLAB function *circ_ vmrnd* (Berens and Velasco, 2009). Frames corresponding to a duration of 50 minutes were clustered together and from all the fluctuations of the individual cells we randomly down sampled 10^5 data points. We then fitted the von Mises distribution to these data, thus estimating mean and variance of the best fit. Data distribution and best fit are shown in S11 Fig C. After that, we performed a Kolmogorov-Smirnov test as a measure of the goodness of the fit, indicating if the initial 10^5 data points belonged to the same distribution as other 10^5 data points randomly sampled from the von Mises distribution. This hypothesis was rejected during the streaming phase ($H = 1$), thus confirming that distribution of the directional fluctuations deviates from the von Mises distribution.

Another feature of criticality commonly investigated is wether cluster size follows a power-law distribution [22]. To study this in our system, we clustered cells that are moving in a similar direction. More specifically, we first coarse-grained the image into 100 squares and computed the average direction of cells inside every square. An adjacency matrix defining the nearest neighbors of every square (left, right,

top, bottom and first elements on the diagonal) was built. We then computed cluster sizes by moving over the nearest neighbors and clustering cells according to their direction, considering cells belonging to the same cluster if their direction differed less than $\pi/5$. As shown in S11 Fig D, before aggregation cells move individually, thus the distribution is skewed on the left, with many small clusters. At a later stage during streaming, cells all move towards the same aggregation center, thus leading to one large cluster with few cells left out. Immediately before streaming we obtain medium-size patches instead, with a cluster-size distribution resembling a power-law.

In order to have a complete characterization of the system, we also considered cell speed as a different mode for which connected correlations can be computed. According to Goldstone's theorem [23, 15], due to breaking of rotational symmetry upon streaming we expect scale-free fluctuations in the transverse direction (soft mode). This is indeed the case for the connected directional correlations as shown in the main text. As cells are always moving we do not expect the same for the speed fluctuations as no symmetry is broken in this case. However, similar to bird flocks in [15] we also see long-range correlations in speed fluctuations. Specifically, using dataset 3 we extracted the velocity modulus of every cell from the tracking algorithm. As done for the directional correlations, also speed correlations were computed by considering a neighborhood radius around every cell, equal to $L/4$ as done in the main text (Fig 3B). The speed correlations were calculated by using Eq. 1. Here, we substituted $\vec{u}_i$ with the velocity modulus of individual cells from which we subtracted the average speed of the neighborhood, i.e. $||\vec{v}_i|| - \frac{1}{N}\sum_{k=1}^{N}||\vec{v}_k||$ [15]. Interestingly, speed correlations exhibit a similar behavior as directional correlations, increasing during streaming (see S12 Fig). This may be due to the fact that we are not dealing with an equilibrium system or to rise in speed after slowing down, which violates momentum conservation.

6 Mutant simulations and comparison with data

Simulation description. To investigate the robustness of aggregation, we perturbed our coarse-grained model in various ways to better understand how cell aggregation is affected in comparison to wild-type simulations (Fig 4 of the main text, movies S10-S14). To measure the effectiveness of aggregation, we considered the correlation length during streaming as a proxy for the range of cell-cell communication and the spatial information of the final cell distribution to represent the quality of aggregation.

Uniform secretion. We first analyzed the case in which cells secrete cAMP without a preferred direction (corresponding to uniform secretion along their contour, and not just secretion from the rear). We found that the correlation length was reduced during streaming. Although the cells were aggregating, they were unable to organize themselves into a single mound, but instead aggregated locally with their nearby neighbors.

Sensing noise. Cell sensing in the wild-type simulations includes some noise, simulated by randomly sampling from a Poisson distribution with average and variance equal to the sensed cAMP concentration. Here, we increased the standard deviation of this distribution tenfold. Surprisingly, aggregation was robust to this noise, with the final aggregation pattern containing just two aggregation centers and the aggregation time even reduced slightly.

Cell-cell adhesion. In terms of cell adhesion, we added a Lennard-Jones potential, which is often used to represent molecular interactions. This potential leads to a strong repulsion at short cell-cell distances to capture volume exclusion (which was implemented to reflect the minimum cell-cell distance in wild-type simulations) and to a medium cell-cell distance attraction, meant to represent cell adhesion. Even though cells came closer to each other during the simulations, this did not result in improved aggregation. Instead, multiple aggregation centers formed.

Asynchronous secretion. Finally, we considered a random refractory period to capture the total absence

of synchronization in the regA mutant [2]. To achieve this in our simulations, each cell was assigned a refractory period randomly sampled from a uniform distribution with the minimum equal to the wild-type refractory period (6 minutes) and the maximum a hundred times longer. As a result, cells fired at different times and consequently there was little self-organization and streaming, although some cells came close together simply due to random movement.

Experimental data analysis We also performed a similar analysis on experimental data of wild-type and mutant cells (regA and rdeA). Specifically, we analyzed cAMP waves and spatial cell distribution from Fig 5 in [24]. The correlations were extracted from the left panels of Fig 5a-c. For this purpose, we used the MATLAB *autocorr* function to measure 1D intensity correlations for all rows and columns of the image. We subsequently computed the average of these to obtain a global measure of correlation. Briefly, this Matlab function computes the autocorrelations of the pixel intensities of a vector with itself for different distances (shifts). As expected, wild-type cells showed a characteristic correlation profile due to the typical spiral waves, which starts from unity when the distance was zero (image perfectly correlated with itself), decreased to become negative to reflect the light stripes, and exhibited some peaks in correspondence to the dark stripes (S13 Fig, blue line). This pattern did not appear in the mutant images (S13 Fig, red and green lines). In order to estimate the correlation length, these correlation profiles were fitted with the function $\exp(-x/x_0)\cos(\omega x)$ to reflect the decreasing correlations and the striped pattern. The largest correlation length x_0 was found for the wild-type cells, while mutant cells exhibited smaller x_0 (see inset of Fig 4 of the main text).

Spatial information was also computed for the central panels in Fig 5a-c in [24], representing cell position during aggregation. Hence, wild-type cells achieved a higher quality of aggregation as compared to mutant cells, suggesting defective aggregation in the latter.

References

[1] Fischer M, Haase I, Simmeth E, Gerisch G, A MT. A brilliant monomeric red fluorescent protein to visualize cytoskeleton dynamics in *Dictyostelium*. FEBS Letters. 2004;577:227.

[2] Gregor T, Fujimoto K, Masaki N, Sawai S. The onset of collective behavior in social amoebae. Science. 2010;328(5981):1021–1025. doi:10.1126/science.1183415.

[3] Sgro AE, Schwab DJ, Noorbakhsh J, Mestler T, Mehta P, Gregor T. From intracellular signaling to population oscillations: bridging size- and time-scales in collective behavior. Molecular Systems Biology. 2015;11(1). doi:10.15252/msb.20145352.

[4] Shannon C. A mathematical theory of communication. Bell System Technical Journal. 1948;27:379.

[5] Shannon C. A mathematical theory of communication. Bell System Technical Journal. 1948;27:623.

[6] Heinz W, Werbin J, Lattman E, Hoh J. Computing Spatial Information from Fourier Coefficient Distributions. The Journal of Membrane Biology. 2011;241(2):59–68. doi:10.1007/s00232-011-9362-x.

[7] Tweedy L, Meier B, Stephan J, Heinrich D, Endres RG. Distinct cell shapes determine accurate chemotaxis. Scientific Reports. 2013;3:2606.

[8] Meinhardt H. Orientation of chemotactic cells and growth cones: models and mechanisms. Journal of Cell Science. 1999;112(17):2867–2874.

[9] FitzHugh R. Impulses and physiological states in theoretical models of nerve membrane. Biophysical Journal. 1961;1(6):445–466.

[10] Nagumo J, Arimoto S, Yoshizawa S. An active pulse transmission line simulating nerve axon. Proceedings of the IRE. 1962;50(10):2061–2070. doi:10.1109/JRPROC.1962.288235.

[11] McMains VC, Liao X, Kimmel AR. Oscillatory signaling and network responses during the development of *Dictyostelium discoideum*. Ageing Research Reviews. 2008;7(3):234–48.

[12] Kriebel PW, Barr VA, Rericha EC, Zhang G, Parent CA. Collective cell migration requires vesicular trafficking for chemoattractant delivery at the trailing edge. The Journal of Cell Biology. 2008;183(5):949–961. doi:10.1083/jcb.200808105.

[13] Bader S, Kortholt A, Van Haastert PJM. Seven Dictyostelium discoideum phosphodiesterases degrade three pools of cAMP and cGMP. Biochemical Journal. 2007;402(1):153–161. doi:10.1042/BJ20061153.

[14] Van Haastert PJM. A stochastic model for chemotaxis based on the ordered extension of pseudopods. Biophysical Journal. 2010;99(10):3345–3354. doi:10.1016/j.bpj.2010.09.042.

[15] Cavagna A, Cimarelli A, Giardina I, Parisi G, Santagati R, Stefanini F, et al. Scale-free correlations in starling flocks. Proceedings of the National Academy of Sciences. 2010;107(26):11865–11870. doi:10.1073/pnas.1005766107.

[16] Lauzeral J, Halloy J, Goldbeter A. Desynchronization of cells on the developmental path triggers the formation of spiral waves of cAMP during Dictyostelium aggregation. Proceedings of the National Academy of Sciences. 1997;94(17):9153–9158.

[17] Endres RG, Wingreen NS. Accuracy of direct gradient sensing by single cells. Proceedings of the National Academy of Sciences. 2008;105(41):15749–54.

[18] Alcantara F, Monk M. Signal propagation during aggregation in the slime mold *Dictyostelium discoideum.* Journal of General Microbiology. 1974;85:321.

[19] Noorbakhsh J, Schwab DJ, Sgro AE, Gregor T, Mehta P. Modeling oscillations and spiral waves in *Dictyostelium* populations. Physical Review E. 2015;91:062711. doi:10.1103/PhysRevE.91.062711.

[20] Skoge M, Meir Y, Wingreen NS. Dynamics of Cooperativity in Chemical Sensing among Cell-Surface Receptors. Physical Review Letters. 2011;107:178101. doi:10.1103/PhysRevLett.107.178101.

[21] Krotov D, Dubuis JO, Gregor T, Bialek W. Morphogenesis at criticality. Proceedings of the National Academy of Sciences. 2014;111(10):3683–3688. doi:10.1073/pnas.1324186111.

[22] Chialvo DR. Emergent complex neural dynamics. Nature Physics. 2010;6(10):744–750.

[23] Goldstone J. Field theories with «Superconductor» solutions. Il Nuovo Cimento (1955-1965). 1961;19(1):154–164. doi:10.1007/BF02812722.

[24] Sawai S, Guan X, Kuspa A, Cox E. High-throughput analysis of spatio-temporal dynamics in *Dictyostelium.* Genome Biology. 2007;8:R144.
