# Supplementary figures and images for: A critical-like collective state leads to long-range cell communication in Dictyostelium discoideum aggregation
Source: PLoS Biol. 2017 Apr 19;15(4):e1002602. doi: 10.1371/journal.pbio.1002602 (PMC5396852; doi:10.1371/journal.pbio.1002602)

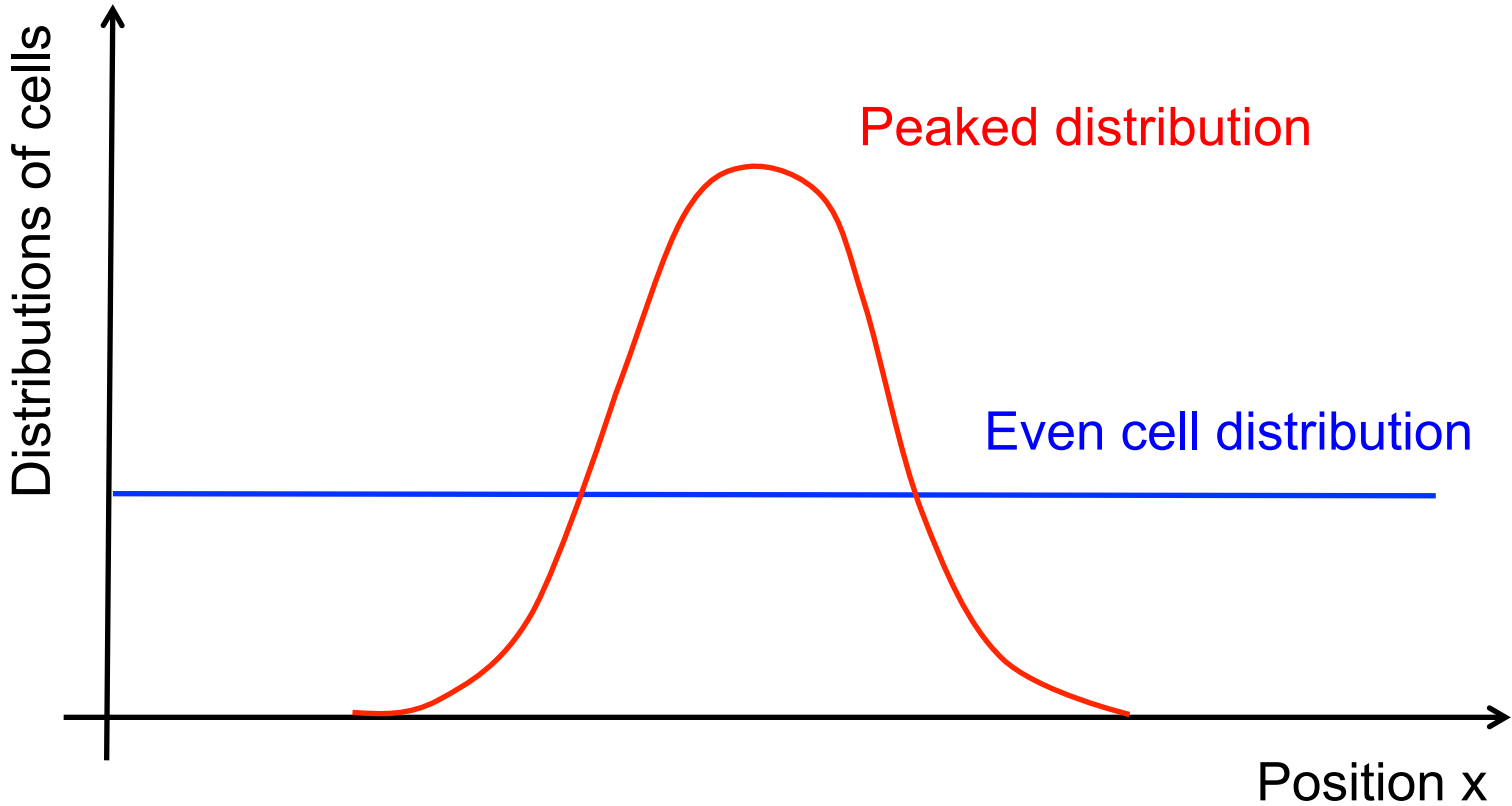

Supplement: S1 Fig — The spatial information of a hypothetical one-dimensional cell distribution (red) can be defined as the “entropy” of a maximally disordered cell distribution (even but random cell distribution in blue) minus the “entropy” of the actually peaked cell distribution (red). The more ordered (peaked or structured) the actual cell distribution, the higher the spatial information, as the entropy (uncertainty where cells are) decreases with increasing order. (PDF) [file pbio.1002602.s002.pdf]

Is there at least one cell here?

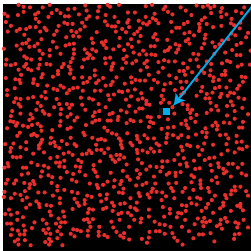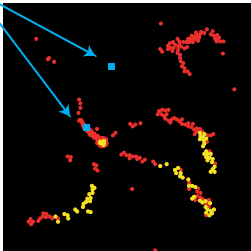

Supplement: S2 Fig — The spatial information can be thought of as a measure of the confidence with which we can predict the presence (or absence) of a cell at a given point. Since cells are initially randomly distributed in our simulations, predicting the presence of a cell in the small light blue square in the panel on the left is much more difficult than predicting the presence of a cell in the two squares in the panel on the right. (PDF) [file pbio.1002602.s003.pdf]

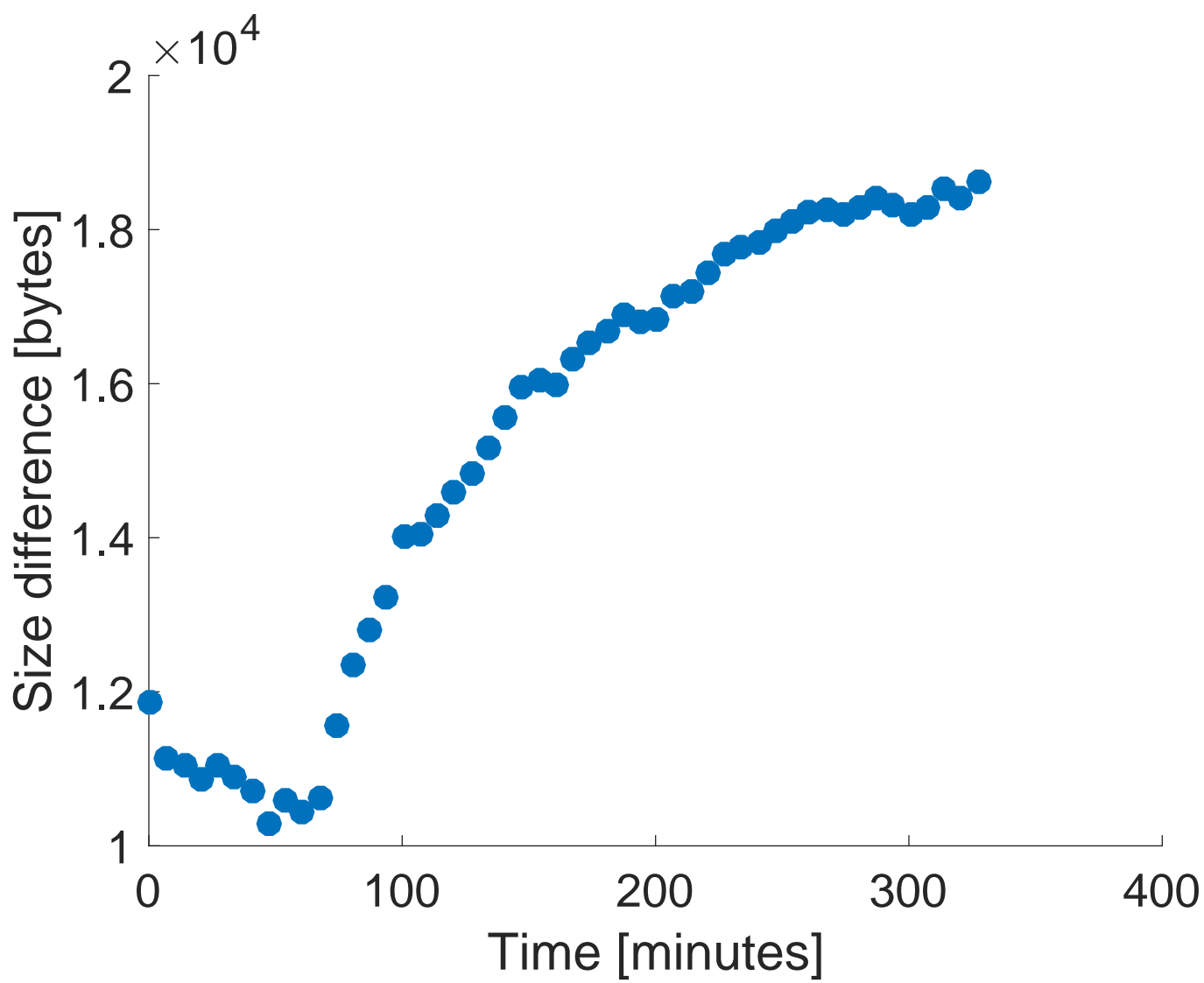

Supplement: S3 Fig — Example of file size difference between the original JPG image and the corresponding zipped image for a coarse-grained wild-type simulation. This difference increases with cell aggregation, similar to the spatial information of the simulation shown in Fig 1F, inset ii of the main text. (PDF) [file pbio.1002602.s004.pdf]

A

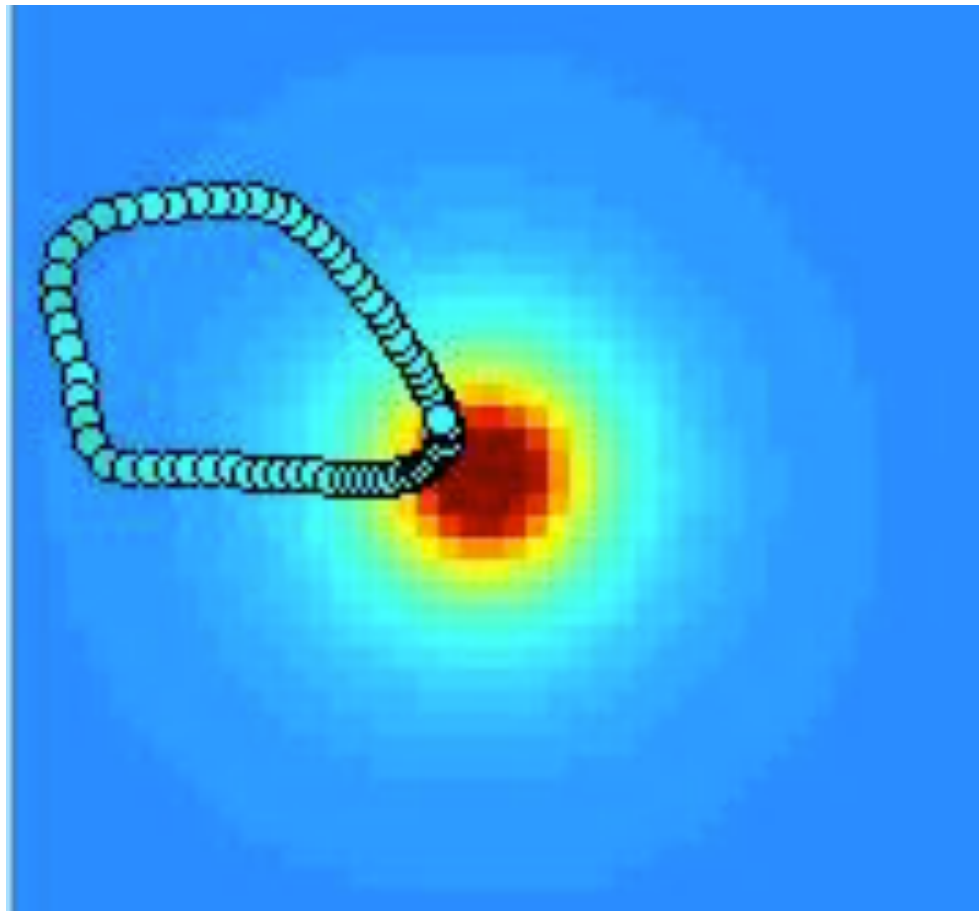

B

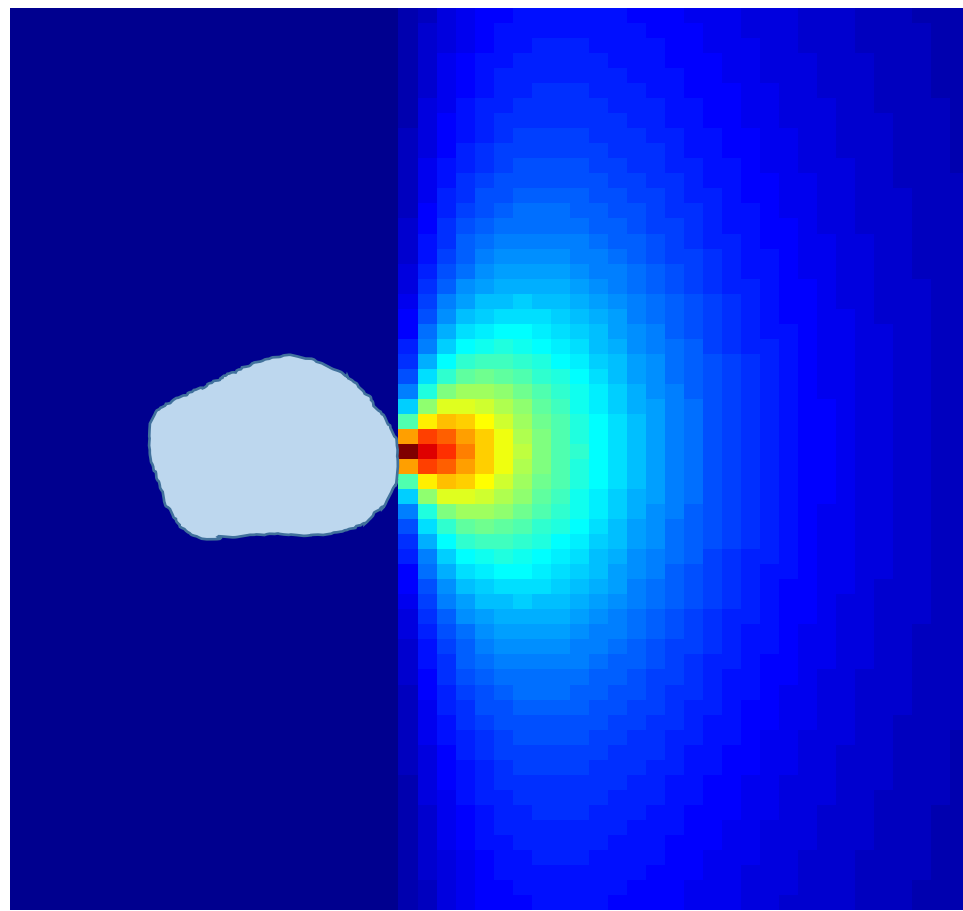

Supplement: S4 Fig — Example of leaked cAMP for a detailed (A) and a coarse-grained (B) simulation. Cell shape was artificially added to an image of coarse-grained simulation in order to facilitate easier comparison (in coarse-grained simulations, cells are point-like objects with volume exclusion). cAMP secretion occurs from the rear of the cell, and in the case of the coarse-grained model, cAMP propagates more easily in the direction opposite to cell motion. (PDF) [file pbio.1002602.s005.pdf]

**A**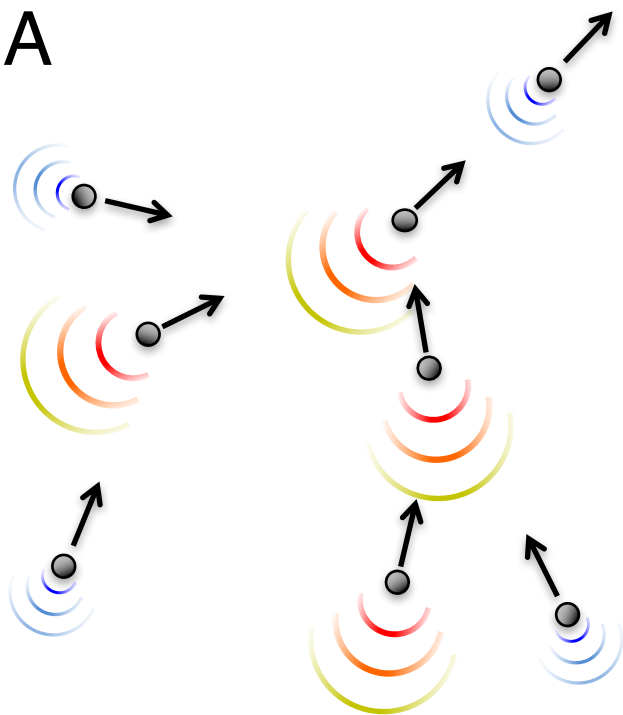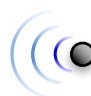 non-firing cell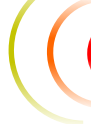 firing cell**B**

cAMP concentration

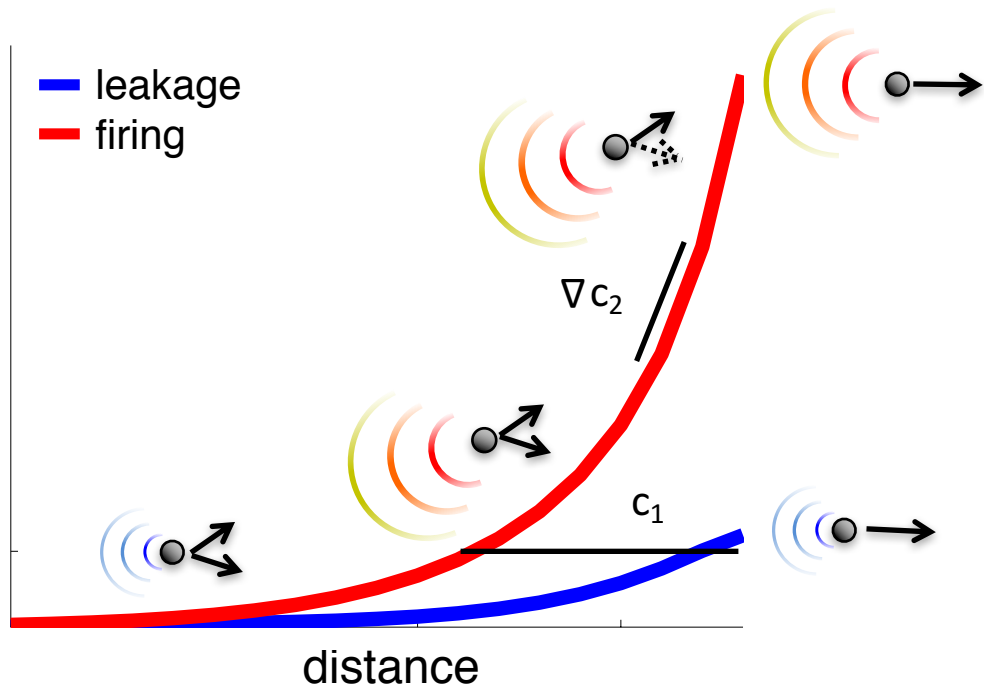

Supplement: S5 Fig — (A) Schematic showing how cells chase each other. (B) Secretion and subsequent decision making. The behavior of cell b on the left depends on the cAMP concentration sensed and hence on the distance from the first cell (x axis) only. If the sensed concentration is above threshold c1, cell b emits a pulse of cAMP. The spatial gradient of cAMP concentration determines the choice of movement. If the gradient is below ∇c2, cell b chooses randomly between the two directions allowed; if instead the gradient is greater than ∇c2, it chooses the direction that maximizes cAMP concentration. (PDF) [file pbio.1002602.s006.pdf]

A

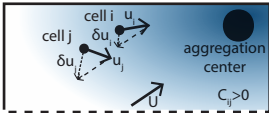

B

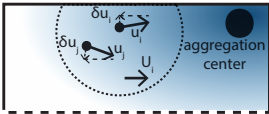

Supplement: S6 Fig — The dashed line at the bottom of the panels represents the fact that only part of the whole image is shown. Cells i and j are moving towards the centre of aggregation, with directions ui and uj. (A) U represents the global average cell direction. δui and δuj are calculated by subtracting average U from vectors ui and uj to represent fluctuations. The resulting directional correlations between i and j calculated with this procedure are greater than zero. (B) The reference direction Ui is now calculated for the neighborhood of cell i and is subtracted from both ui and uj. Fluctuations δui and δuj are now correctly anticorrelated. (PDF) [file pbio.1002602.s007.pdf]

**A**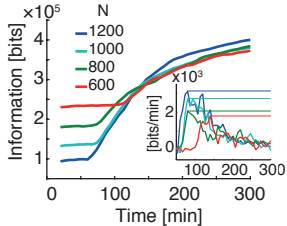**B**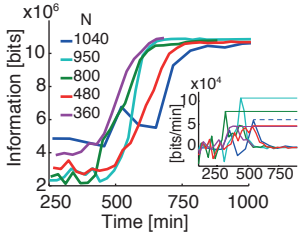**C**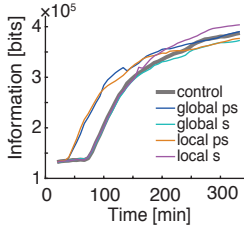

Supplement: S7 Fig — (A) Spatial information changes with n in in silico data. (inset) Time derivative of spatial information profiles. The change in spatial information is larger for higher cell numbers as indicated by the horizontal lines corresponding to the peak values. (B) Corresponding spatial information as a function of time for the experimental data. (inset) Similar to the simulations, the derivative tends to have higher peaks for experiments with higher cell densities, although the dark blue line does not match the trend. (C) Effect of perturbations on the system during aggregation compared to control without perturbations. A speeding up of aggregation is seen if a localized or a global, spatially uniform pulse of cAMP is given to the system during prestreaming (ps). No effect on aggregation speed is noticed if the system is perturbed during streaming (s). See S6–S9 Movies. Numerical values for simulations and experimental results are reported in S17 and S18 Data, respectively. (PDF) [file pbio.1002602.s008.pdf]

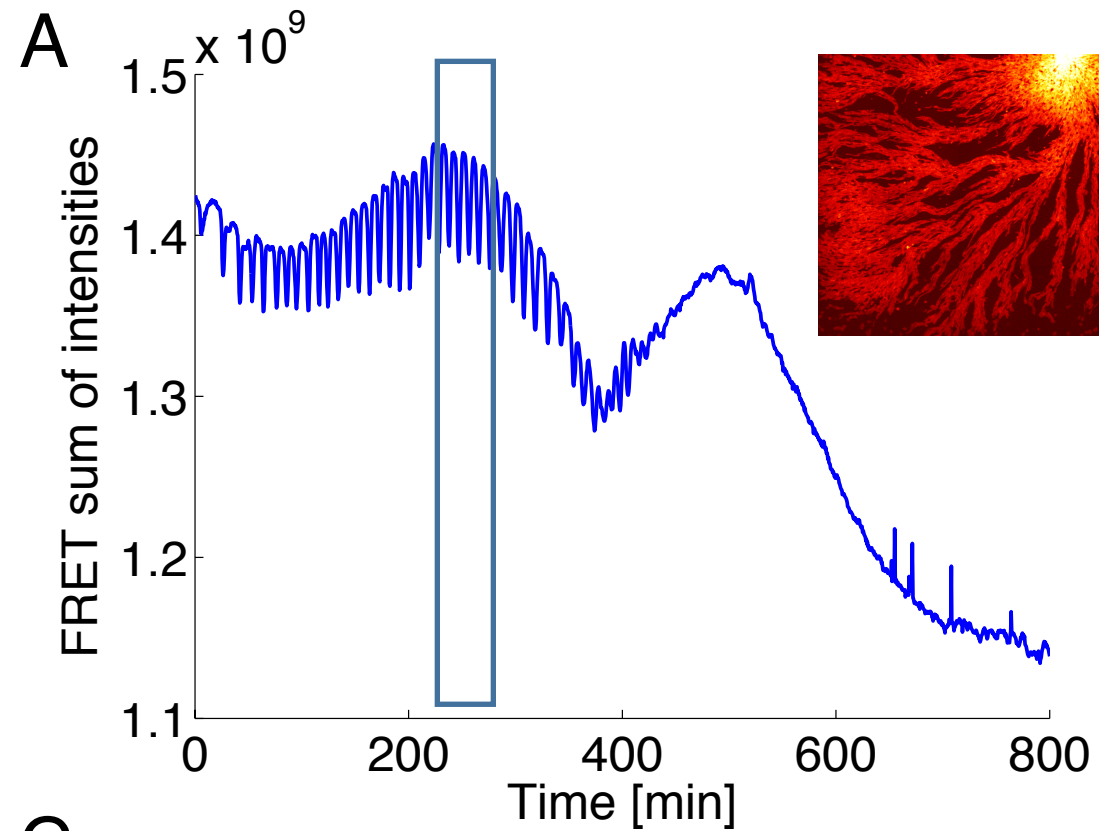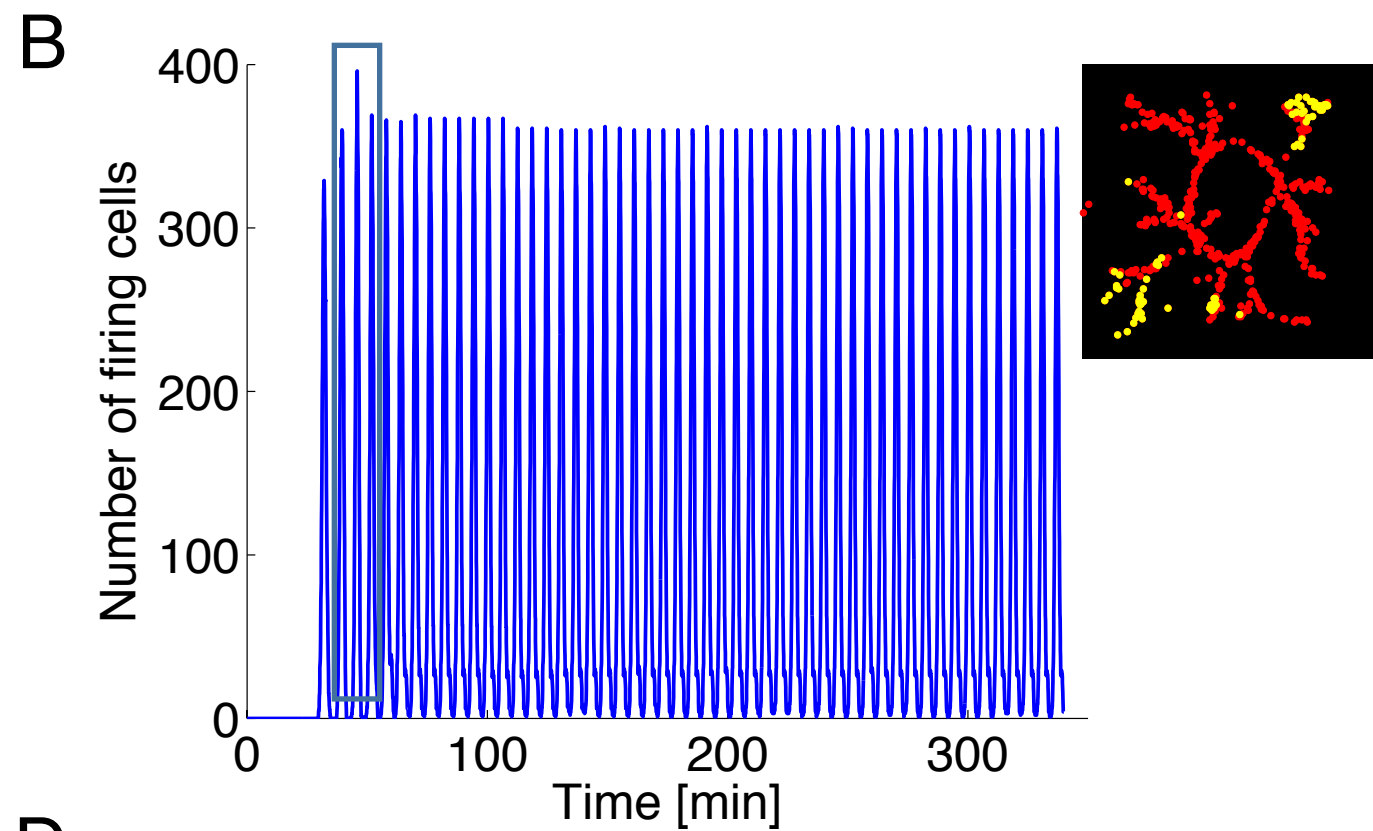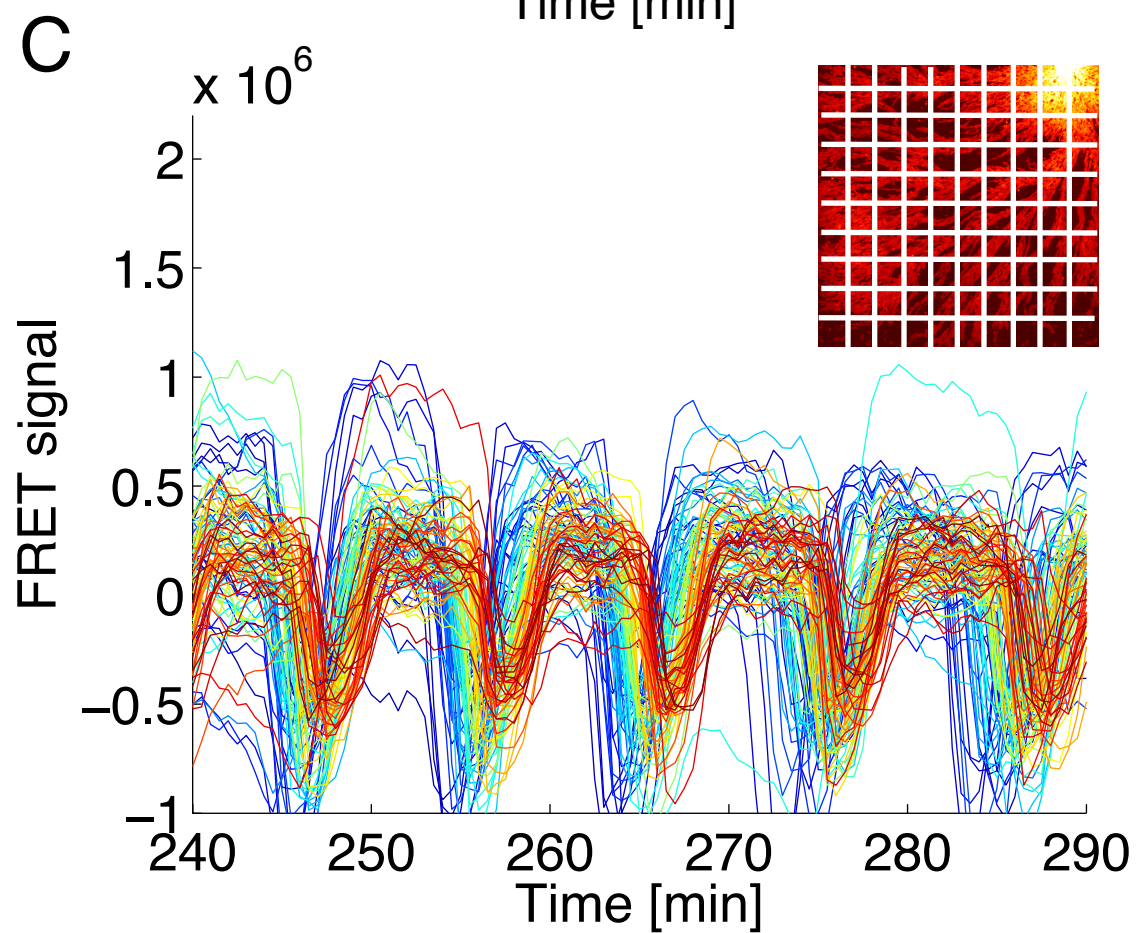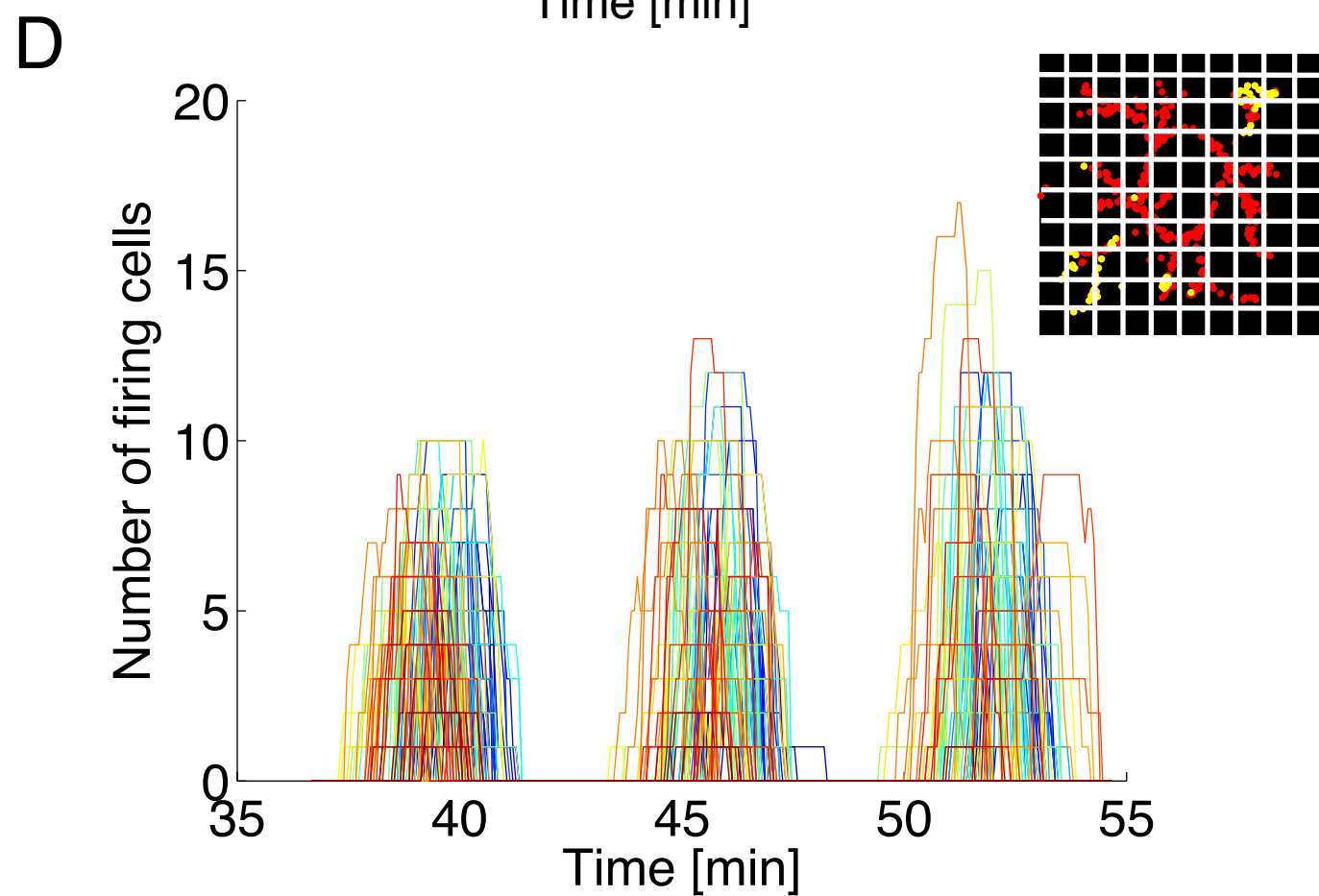

Supplement: S8 Fig — (A) FRET signal (sum of the intensities of all pixels) in time for dataset 3. (B) Number of firing cells in time for a simulation with n = 500 cells. (C) Spatial propagation of cAMP pulses. The sum of the FRET signal of 100 squares is shown (see inset for the 100 regions). Data were processed with a moving average filtering for better visualization. Note secretion of cAMP corresponds to a decrease in FRET signal. Wave-like propagation of cAMP in space can clearly be seen. (D) Analysis of (C) performed on simulated data. Similar to the data, the signal propagates in space with a small delay between firing cells. Numerical data are provided in S19 Data. (PDF) [file pbio.1002602.s009.pdf]

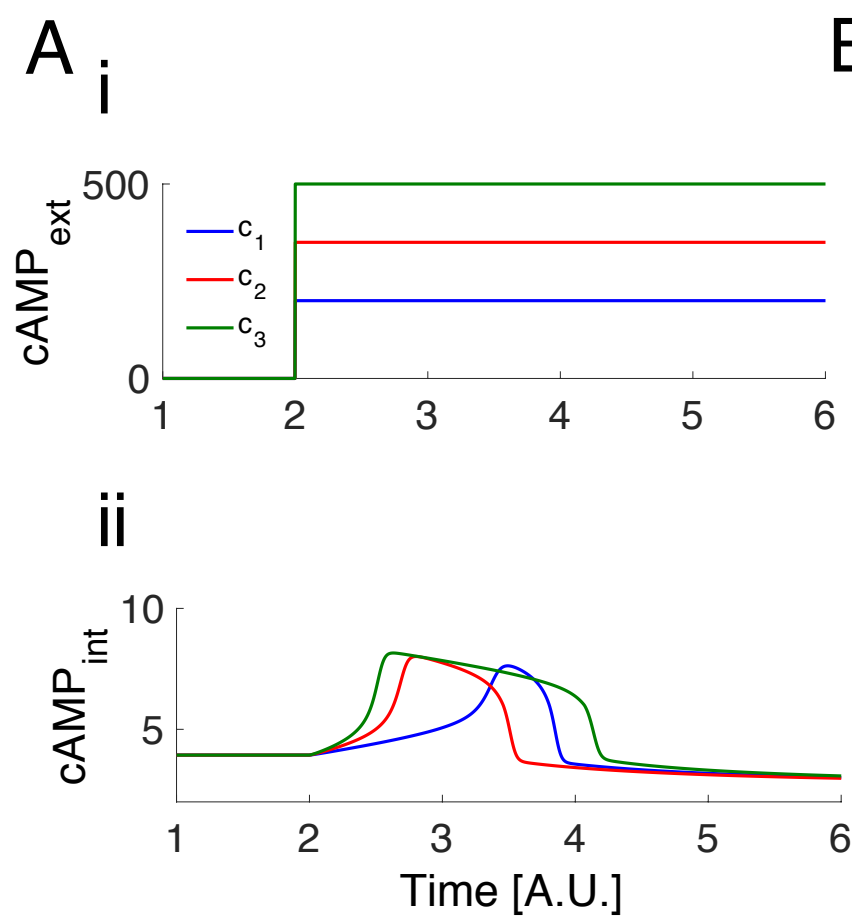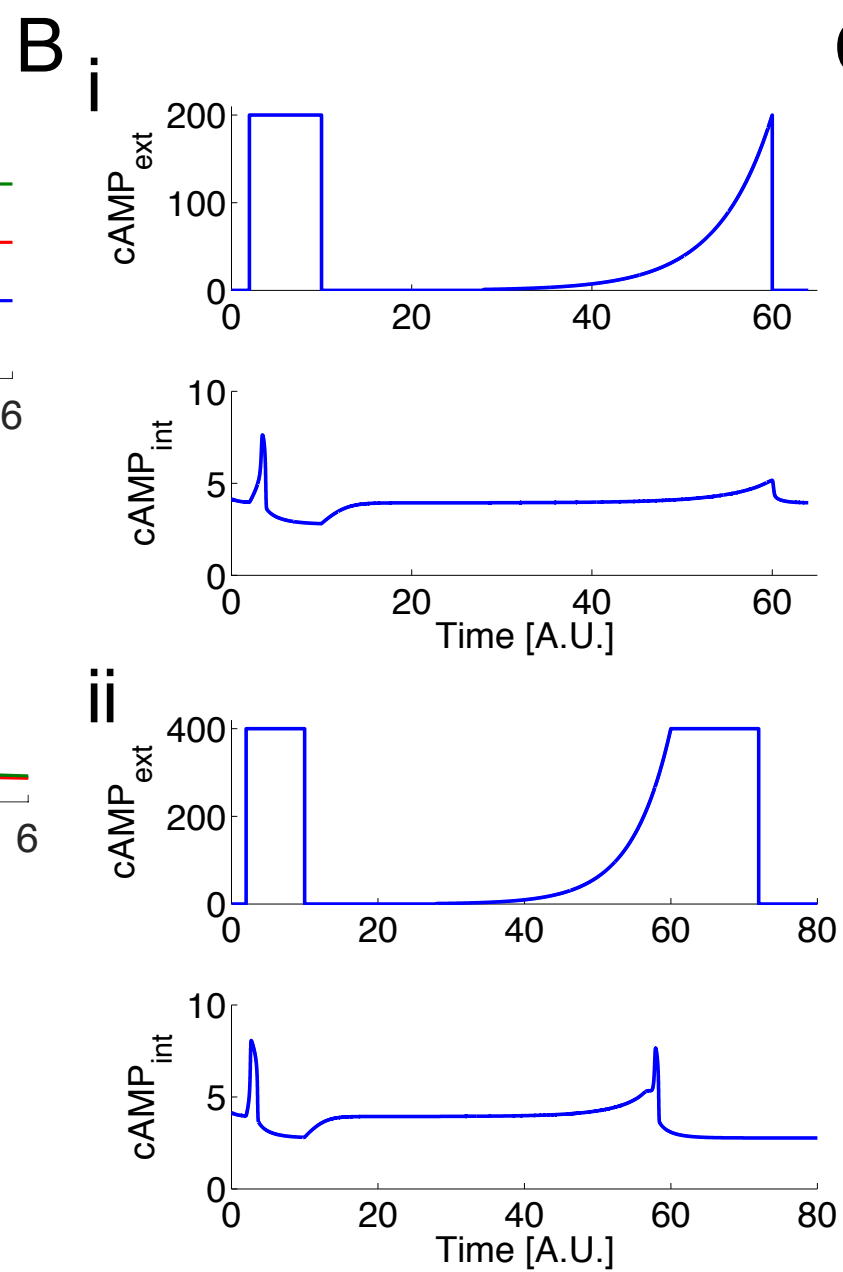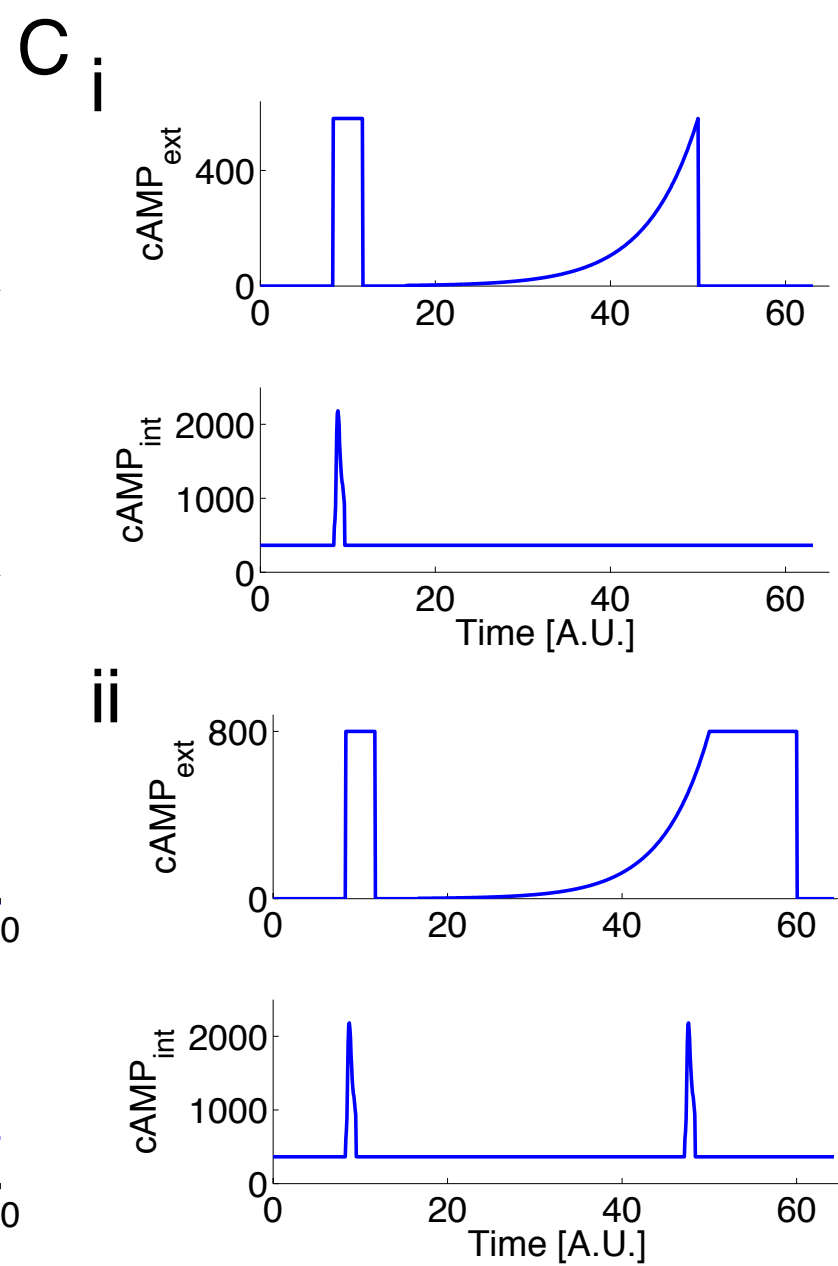

Supplement: S9 Fig — (A) Detailed model responses (ii) to increasing concentrations of external cAMP (i) (amplitudes are 200, 350, and 500). (B) Response of FitzHugh—Nagumo module of detailed model changes depending on the rate of external cAMP for small-amplitude (i) and large-amplitude (ii) stimulus. (C) Response of coarse-grained model to different cAMP rates for small (i) and high (ii) concentrations. Numerical data are provided in S20 Data. (PDF) [file pbio.1002602.s010.pdf]

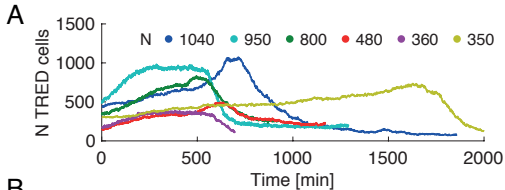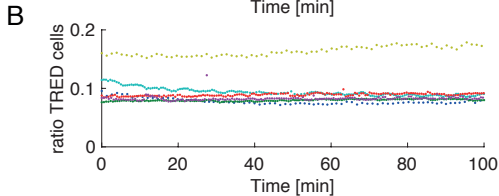

Supplement: S10 Fig — (A) Number of TRED cells, estimated by segmentation. In order to avoid boundary artifacts, a fraction of the field of cells was imaged. This implies that during aggregation, the number of cells increases because of movement towards the aggregation center. In particular, the number of cells increases during streaming (while after aggregation the number decreases again because of difficulty in following individual cells). (B) Fraction of TRED cells with respect to total cells for the first 100 min. Numerical data are provided in S21 Data. (PDF) [file pbio.1002602.s011.pdf]

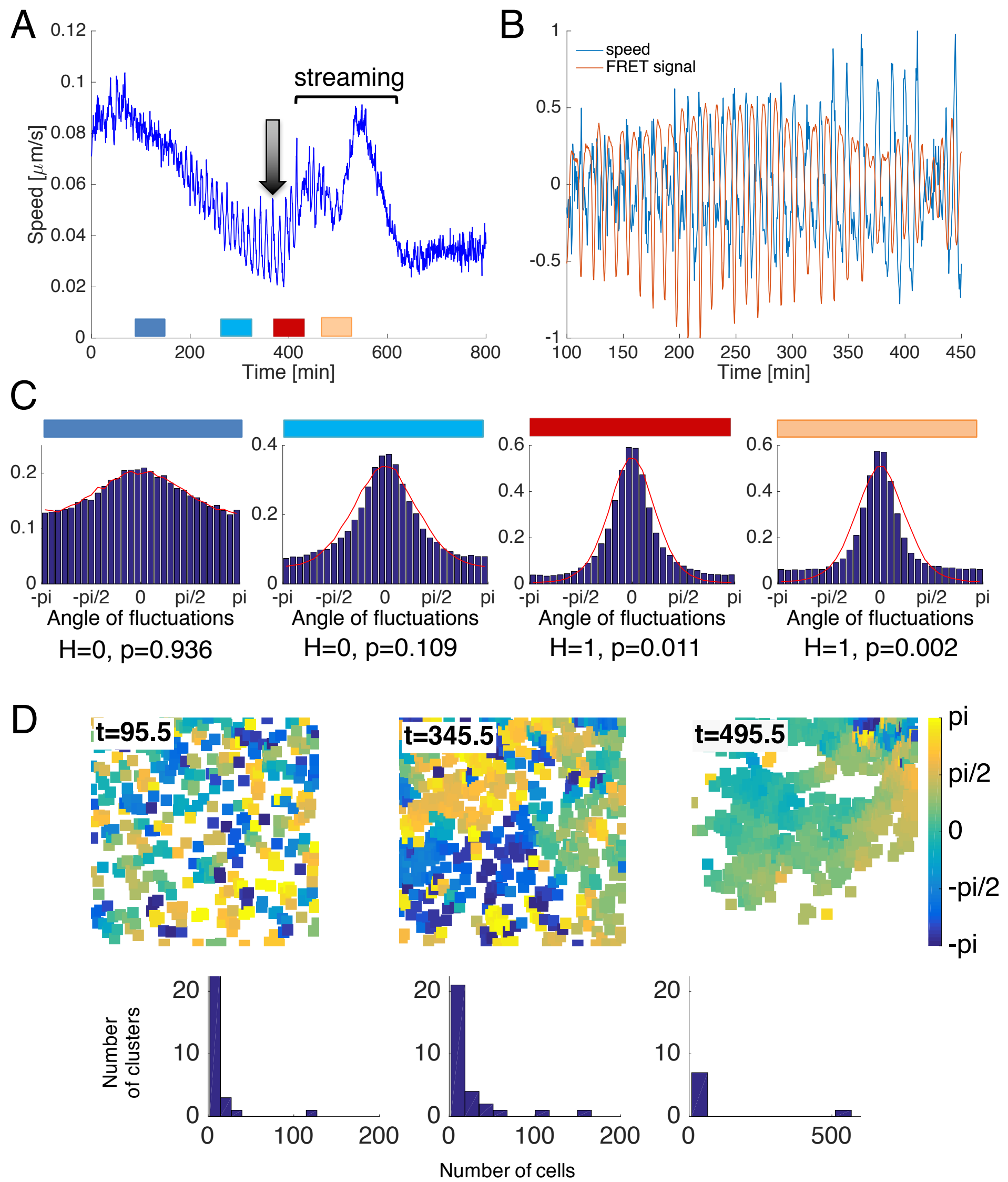

Supplement: S11 Fig — (A) Cell speed during aggregation. Speed was estimated from the average cell movement from frame to frame. (B) Speed-FRET oscillations. A moving average filter was applied to the two signals, which were subsequently normalized for comparison. (C) Distributions of directions. The different angles of directional fluctuations were collected for 50 frames (see color bar in panel A for corresponding times) and plotted as a distribution. The red lines represent the fits to the von Mises distribution. Underneath the plots, the results of a two-sample Kolmogorov—Smirnov test performed with MATLAB kstest2 function and corresponding p -values are reported. H = 0 (H = 1) indicates that the null hypothesis of identical distributions cannot (can be) rejected. (D) Cluster-size distribution for different time points. (top) Cell directions. Squares represent the angles of movement of individual cells. (bottom) Corresponding cluster-size distributions. Numerical data are provided in zipped folder S22 Data. (PDF) [file pbio.1002602.s012.pdf]

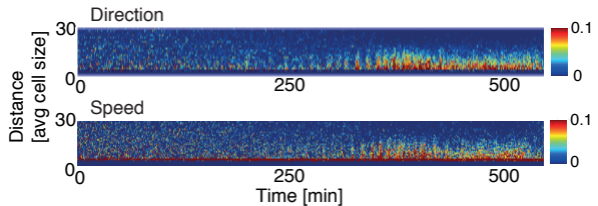

Supplement: S12 Fig — Connected correlations increase with similar amplitudes and timings for direction (already presented and described in the main text) and speed. Numerical data are provided in S13 Data. (PDF) [file pbio.1002602.s013.pdf]

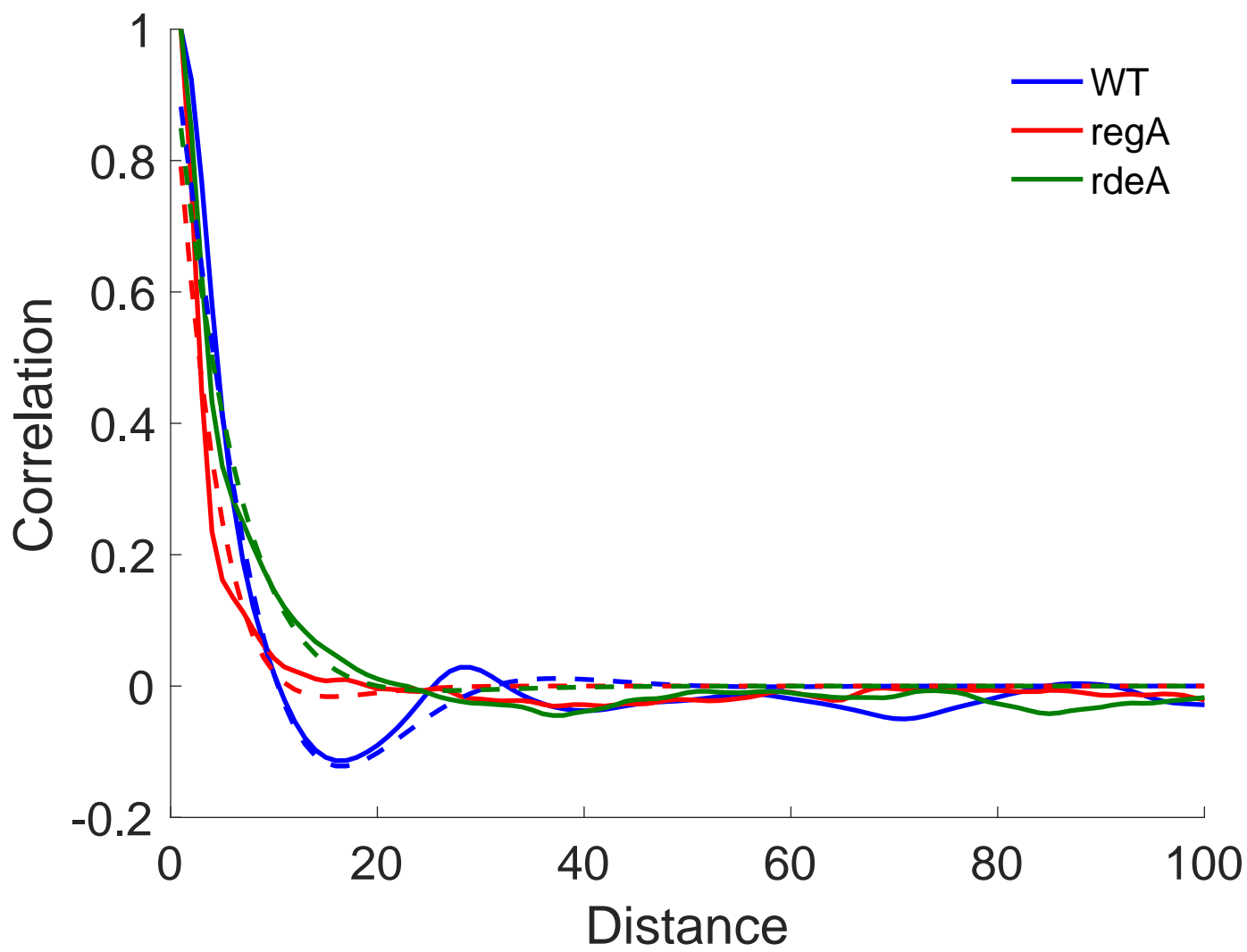

Supplement: S13 Fig — Autocorrelations (solid lines) and corresponding fits (dashed lines) for the left panels of Fig 5A–C in [55]. Numerical data are provided in S16 Data. (PDF) [file pbio.1002602.s014.pdf]
