# Supplementary material for: A critical-like collective state leads to long-range cell communication in Dictyostelium discoideum aggregation
Source: PLoS Biol. 2017 Apr 19;15(4):e1002602. doi: 10.1371/journal.pbio.1002602 (PMC5396852; doi:10.1371/journal.pbio.1002602)
Supplement: S1 Table — Space and time units were set according to the diffusion constant of cAMP, and are respectively μm and s for every parameter. Concentration is instead in arbitrary units but can be chosen of order μM for the intracellular and nM for extracellular cAMP [22]. (PDF) [file pbio.1002602.s015.pdf]

k_1	11.3	k_2	75	k_3	75
k_4	6.6	k_5	0.66	k_6	1238
k_7	0.25	k_8	250	k_9	3
D_1	250 [1]	D_2	62.5		

References

[1] Lauzeral J, Halloy J, Goldbeter A. Desynchronization of cells on the developmental path triggers the formation of spiral waves of cAMP during Dictyostelium aggregation. Proceedings of the National Academy of Sciences. 1997;94(17):9153–9158.
